# A case of successful endocardial ablation for the epicardial posteroseptal accessory pathway using open-window mapping combined with the extended early-meets-late algorithm

**DOI:** 10.1016/j.hrcr.2025.05.006

**Published:** 2025-05-11

**Authors:** Masaya Watanabe, Tadafumi Nanbu, George Suzuki, Akihiko Yotsukura, Izuni Yoshida, Masayuki Sakurai

**Affiliations:** 1 Department of Cardiology, Hokko Memorial Hospital, Sapporo City, Japan; 2 Department of Cardiovascular Medicine, Hokkaido University Graduate School of Medicine, Sapporo, Japan

**Keywords:** Open-window mapping, Epicardial accessory pathway, Extended early-meets-late, Catheter ablation, Electroanatomical mapping

**Key Teaching Points**

• Ablation of epicardial accessory pathway (AP) presents significant challenges and often requires intervention within the coronary sinus system or epicardial access, and the conventional mapping method failed to identify the suitable ablation point at the endocardial side in this case.
• Open window mapping combined with the extended early-meets-late algorithm enables precise identification and ablation of epicardial posteroseptal APs at the endocardial peritricuspid area.
• At the extended early-meets-late gap, the sharpest local signals are based on the maximum rate of voltage change in the later phase of the QRS complex, which could reflect a part of epicardial AP conduction.

## Introduction

Catheter ablation of epicardial posteroseptal accessory pathways (APs) often requires applications within the coronary sinus (CS) or middle cardiac vein (MCV), and in rare cases, an epicardial approach may be necessary, making these ablations challenging.^1^ Open-window mapping (OWM) is a novel technique that enables continuous high-density mapping of both the atria and ventricles and has been shown to reduce misannotation.^2^ In addition, adding the extended early-meets-late (EEML) algorithm provides the operator with a locality visual for proximal and distal insertion sites of the AP, leading to reduced mapping and ablation time compared with the conventional mapping.^3^ However, to date, the efficacy of OWM for epicardial AP ablation has not been fully explored. Here, we present a case of an epicardial AP that was successfully visualized and ablated from the endocardial tricuspid annulus using OWM combined with the EEML algorithm.

## Case report

**Figure 1:**
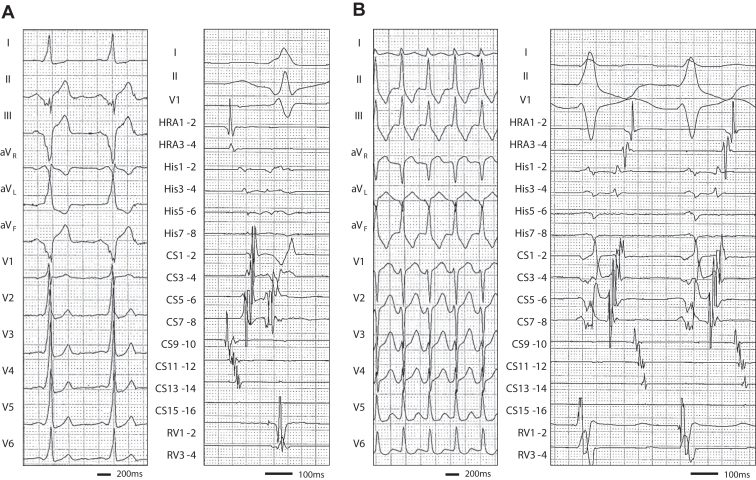
**A:** Twelve-lead electrocardiogram (left panel) and intracardiac electrogram (right panel) during sinus rhythm. **B:** Twelve-lead electrocardiogram (left panel) and the intracardiac electrograms (right panel) recorded during orthodromic atrioventricular tachycardia. CS = coronary sinus; HRA = high right atrium; MCV = middle cardiac vein; RV = right ventricle.

**Figure 2:**
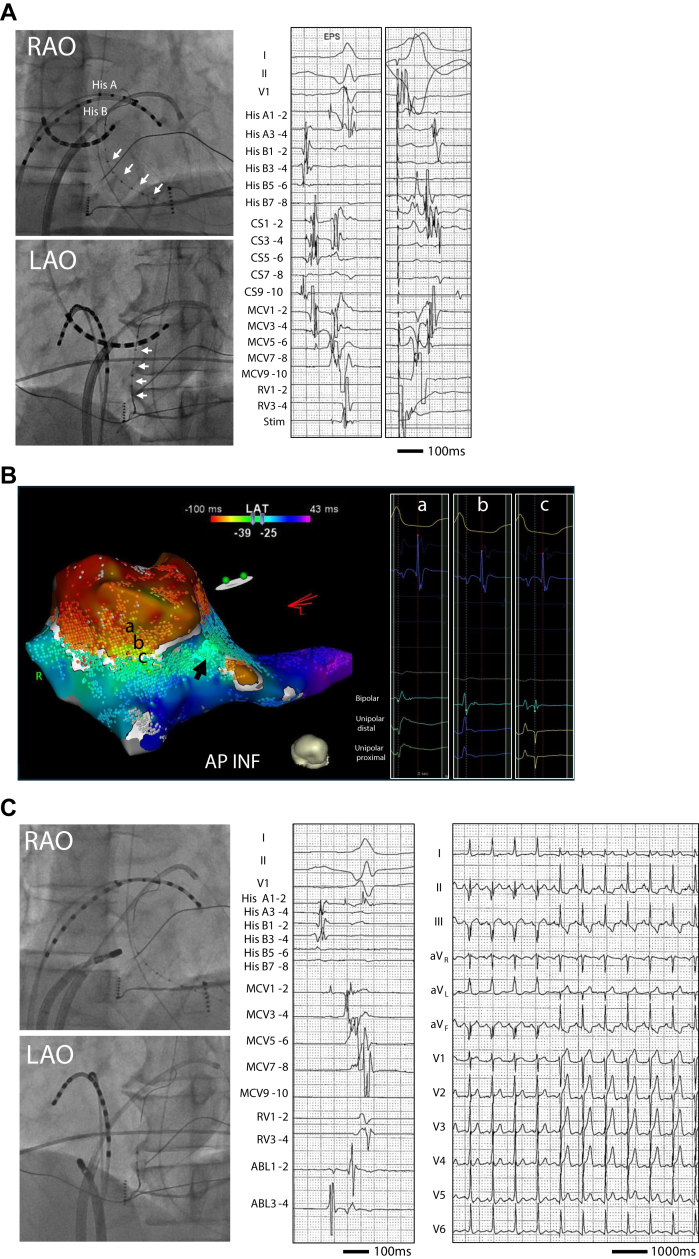
**A:** Fluoroscopic images (*upper and lower left panels*) and intracardiac electrograms (EGM) recorded during sinus rhythm (*left tracing*) and right ventricular pacing (*right tracing*). A 2.7 F over-the-wire-type decapolar catheter (EPstar Fix AIV; Japan Lifeline, Tokyo, Japan) was introduced into the lateral branch of the CS through the internal mammary artery guiding catheter (Mach1; Boston Scientific Corporation, Marlborough, MA) and retrogradely advanced into the middle cardiac vein (*white arrows*). **B:** Open-window mapping with the extended early-meets-late algorithm set at 30% during orthodromic atrioventricular tachycardia. The extended early-meets-late gap was observed in the peritricuspid annulus at the 6 o’clock position. The *black arrow* indicates atrial excitation from the ostial CS. The EGMs in the right column correspond to recordings at each mapped location. It is of note that the greatest slope of the maximum rate of voltage change, which reflects the local activation time, was observed at the first negative slope of the unipolar negative deflection during ventricular activation at point “a.” In contrast, at point “b,” the greatest slope of maximum rate of voltage change was recorded at the downward slope of the positive deflection in the later phase of the QRS complex. **C:** Fluoroscopic images (left panels) and EGMs at the successful ablation site before (middle panel) and electrocardiogram during radiofrequency application (right panel). Radiofrequency application (35 W) eliminated the delta wave in 6 seconds. CS = coronary sinus; LAO = left anterior oblique; MCV = middle cardiac vein; RAO = right anterior oblique; RV = right ventricle.

A 45-year-old man with recurrent palpitations, previously diagnosed as having Wolff-Parkinson-White syndrome, was referred to our hospital for catheter ablation. Electrocardiography during sinus rhythm showed a delta wave with positive deflection in lead I and negative deflections in all inferior leads, along with an R/S ratio of < 1 in lead V1, suggesting an epicardial AP near the posterior septum (Figure 1A). Contrast-enhanced computed tomography revealed no anatomic abnormalities of the CS system. After obtaining an informed consent, a cardiac electrophysiological study using a high-density mapping system CARTO 3 mapping system (Biosense Webster Inc, Diamond Bar, CA) was performed under deep sedation. During sinus rhythm, the intracardiac electrogram showed an HV interval of 18 ms, with the earliest ventricular activation observed at the CS ostium (Figure 1A). Orthodromic atrioventricular tachycardia via the posteroseptal AP was easily induced by programmed stimulation (Figure 1B). Electrode catheters were subsequently positioned in the CS and MCV, along with those in the His bundle and the right ventricle. Both antegrade and retrograde excitations via the AP were evaluated. Although antegrade ventricular excitation occurred simultaneously at the ostial CS and MCV, retrograde atrial excitation during right ventricular pacing was slightly earlier in the ostial CS than in the MCV (Figure 2A). Given that epicardial APs are often treatable within the CS system, radiofrequency (RF) applications were delivered from the MCV (at a power setting of 10 W) and ostial CS (25 W) using an irrigation catheter (Navistar STSF, Biosense Webster Inc). However, the impedance immediately increased, and RF application could not be continued. We subsequently mapped the atrioventricular tachycardia using OWM combined with the EEML algorithm using the Octaray mapping catheter (Biosense Webster Inc) (Figure 2B and Supplemental Video 1). The earliest atrial excitation was identified in 2 areas: the ostial CS (*black arrow*) and the region near the tricuspid annulus at the 6 o’clock position. With the EEML threshold at 30%, the EEML gap was localized at the 6 o’clock position of the tricuspid annulus. RF application (35 W) at the tricuspid annulus successfully eliminated the AP (Figure 2C). Neither antegrade nor retrograde conduction via the AP was observed after the ablation. The patient has remained free from arrhythmia recurrence for 3 months.

## Discussion

Ablation of epicardial AP presents significant challenges and often requires intervention within the CS system. In some cases, an epicardial approach may also be necessary. In the present case, the earliest atrial excitation was observed near the tricuspid annulus at the 6 o’clock position and at the ostial CS. In addition, an isoelectric period was recorded at the successful ablation site. These observations suggest that the AP was relatively distant from the myocardium mappable from the endocardium or the CS system. From an anatomic standpoint, it would be likely that connecting musculature overlying the CS and ventricle was present,^1^ which could explain why the earliest atrial activation was recorded at 2 relatively distant sites. Owing to their anatomic proximity, most epicardial posteroseptal APs are ablated using a coronary venous approach.^1^ However, it is important to recognize that ablation within the CS carries potential procedural risks, including conduction block and CS injury, and limitations in current delivery owing to elevated impedance, as observed in our case. Therefore, preoperative electrocardiographic diagnosis and precise mapping, including the peritricuspid annulus, are essential for successful epicardial posteroseptal AP ablation.

OWM is a novel technique that uses a high-density 3-dimensional mapping system, offering a sufficiently broad window of interest to include both atrial and ventricular electrograms.^2^ This system automatically annotates the sharpest local signals based on the maximum rate of voltage change (dV/dt), regardless of whether the signals originate from the atrium, ventricle, or AP.^2^ OWM has been reported to provide higher accuracy in identifying the location of accessory conduction pathways than conventional point-by-point mapping. Moreover, it is expected to reduce the mapping time and number of energy applications.^2^^,^^4^ In previous studies demonstrating the efficacy of OWM, many patients had left- or right-sided atrioventricular annuli, where continuous fractionated potentials were often recorded at successful ablation sites.^5^ However, in cases of epicardial AP, fractionated potentials are frequently absent, necessitating more precise mapping to eliminate AP conduction from the endocardial side.

**Figure 3:**
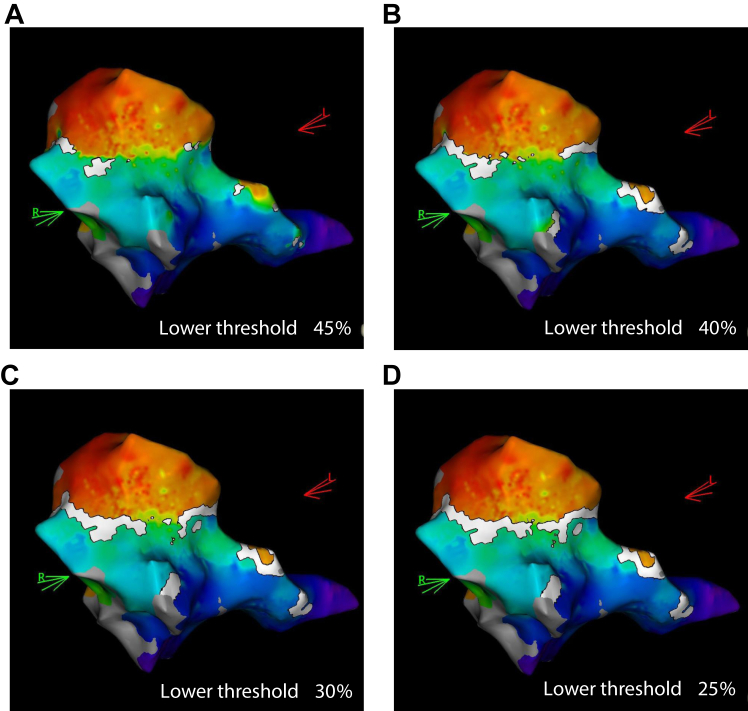
Pictures of the open-window mapping with different thresholds of the extended early-meets-late algorithm: **A:** 45%, **B:** 40%, **C:** 30%, and **D:** 25%.

Although OWM can be performed using other mapping systems, the EEML algorithm is a specific feature of the CARTO mapping system. This system automatically assesses the timing between 2 adjacent local activation points. The timing difference is calculated and expressed as a percentage of the total mapped cycle length. When the excitation time between adjacent points exceeds a certain threshold, white lines indicating possible local conduction block are displayed, providing the operator with a visual representation of the proximal and distal AP insertion sites.^3^^,^^5^ A visual demonstration of how white lines (representing presumed conduction block) appear when the EEML threshold is adjusted is presented in Figure 3. Thus, when fractionated AP potentials are absent, the OWM combined with the EEML algorithm would be particularly useful.

In our case, OWM showed that the greatest slope of dV/dt, which reflects the local activation time, was observed at the first negative slope of the unipolar negative deflection during ventricular activation at point “a” (Figure 2B). In contrast, at point “b,” the greatest slope of dV/dt was recorded at the downward slope of the positive deflection in the later phase of the QRS complex, which could reflect a part of epicardial AP conduction. As a result of such annotation using OWM at point “b,” an EEML gap reflecting conduction through the AP would have been visualized at the tricuspid annulus. The novelty of the OWM technique is highlighted when compared with the conventional method, where the window of interest during atrial activation failed to localize the AP (Supplemental Figure 1 and Supplemental Video 2).

To the best of our knowledge, this is the first case demonstrating accurate visualization of the peritricuspid EEML gap in the epicardial posteroseptal AP, which enabled successful endocardial ablation. Our case highlights the effectiveness of combining OWM with the EEML algorithm for this challenging disease.

## Disclosures

The authors have no conflicts of interest to disclose.
