# Supplementary material for: A case of successful endocardial ablation for the epicardial posteroseptal accessory pathway using open-window mapping combined with the extended early-meets-late algorithm
Source: HeartRhythm Case Rep. 2025 May 11;11(7):702–6. doi: 10.1016/j.hrcr.2025.05.006 (PMC12432980; doi:10.1016/j.hrcr.2025.05.006)
Supplement: Supplementary Material [file mmc3.docx]

**Supplementary material**

**A case of successful endocardial ablation for the epicardial posterior-septal accessory pathway using open window mapping combined with the extended early-meets-late algorithm**

Masaya Watanabe; Tadafumi Nanbu; George Suzuki; Akihiko Yotsukura; Izuni Yoshida; Masayuki Sakurai

**Supplementary Figure 1**

Local activation time mapping during orthodromic atrioventricular reentrant tachycardia (AVRT). The window of interest was defined from the onset to the completion of visible atrial activation.


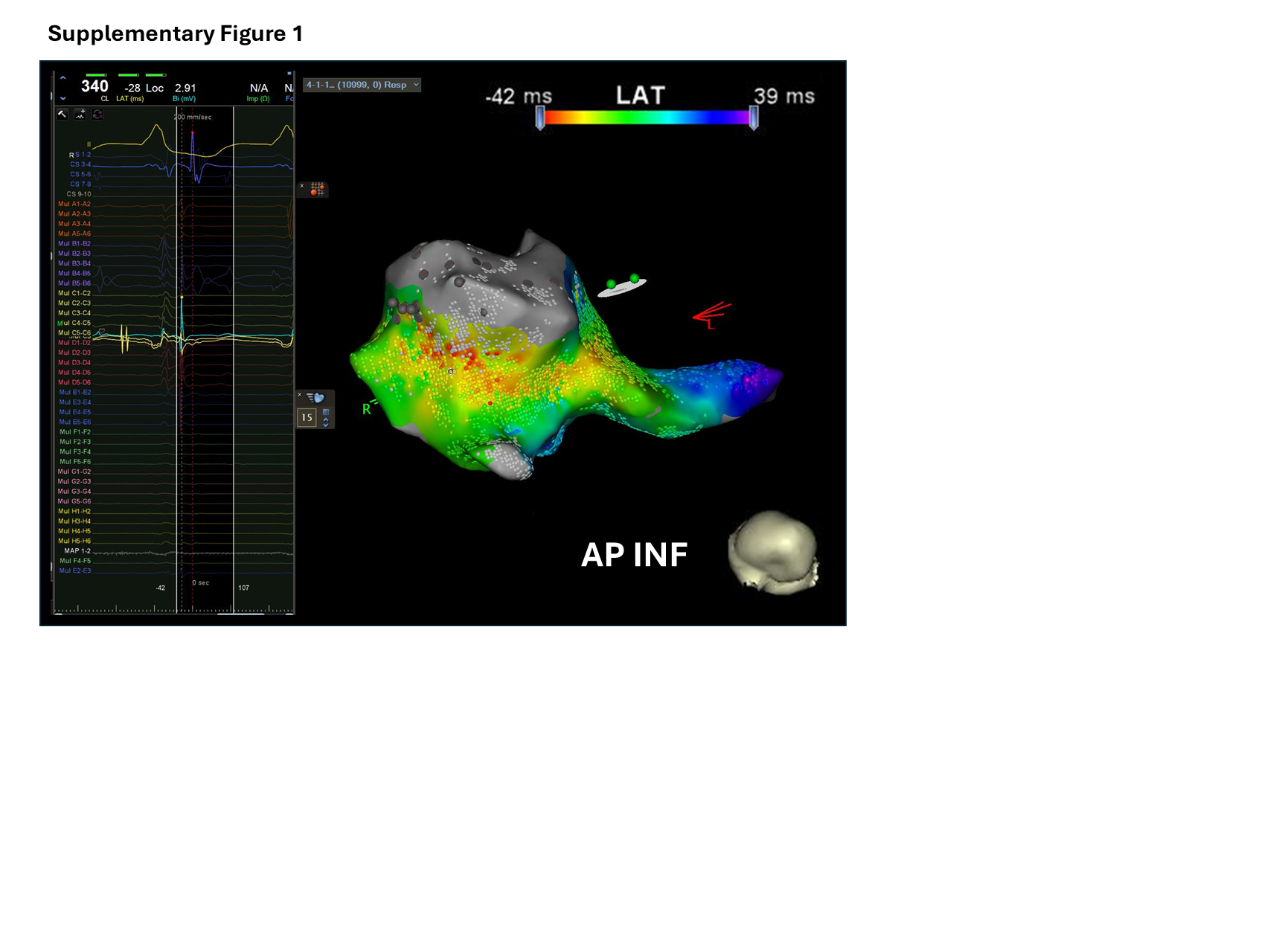


**Supplementary Video S1**

The video showing the propagation map of the open-window mapping (OWM) with the extended early-meets-late (EEML) algorithm during the orthodromic atrioventricular tachycardia (AVRT).

**Supplementary Video S2**

The video showing the propagation map of the conventional local activation time (LAT) mapping during the orthodromic atrioventricular reentrant tachycardia (AVRT). The window of interest was set at the beginning and the end of the visible atrial activation.
